# Supplementary material for: Small Fiber Neuropathy in Sarcoidosis
Source: Pathophysiology. 2021 Dec 20;28(4):544–50. doi: 10.3390/pathophysiology28040035 (PMC8830461; doi:10.3390/pathophysiology28040035)
Supplement: Supplementary file 1 [file pathophysiology-28-00035-s001.zip › pathophysiology-1468353-supplementary.pdf]

Table S1. Small fiber neuropathy screening list questionnaire (19).

**Part 1:**	
**These questions are aimed at finding out how often you experience the following complaints.**	
1. I have painful arms[5]	never/sometimes/variably/often/always
2. I suffer from palpitations[1]	never/sometimes/variably/often/always
3. I have problems with my bowel movements[2]	never/sometimes/variably/often/always
4. I have difficulties with urinating (either in emptying my bladder or being able to hold my water) [3]	never/sometimes/variably/often/always
5. My food does not seem to go down well[2]	never/sometimes/variably/often/always
6. I suffer from muscle cramps[4]	never/sometimes/variably/often/always
7. My feet and/or hands are colder than I am used to[5]	never/sometimes/variably/often/always
8. I have chest pain[4]	never/sometimes/variably/often/always
**Part 2:**	
**These questions are aimed at finding out how serious your complaints are.**	
9. I have the feeling that my food gets stuck in my throat[2]	not at all/slightly/variably/moderately/seriously
10. At night I throw the bedclothes off my legs[5]	not at all/slightly/variably/moderately/seriously
11. I have difficulties with urinating (either emptying my bladder or being able to hold my water) [3]	not at all/slightly/variably/moderately/seriously
12. I have dry eyes [6]	not at all/slightly/variably/moderately/seriously
13. I have blurred vision[6]	not at all/slightly/variably/moderately/seriously
14. I feel dizzy when I get up[1]	not at all/slightly/variably/moderately/seriously
15. I have sudden hot flushes[5]	not at all/slightly/variably/moderately/seriously
16. My feet and/or hands are colder than I am used to[5]	not at all/slightly/variably/moderately/seriously
17. I have painful arms[5]	not at all/slightly/variably/moderately/seriously
18. The skin of my legs is over-sensitive[5]	not at all/slightly/variably/moderately/seriously
19. I have a tingling sensation in my hands (pins and needles) [5]	not at all/slightly/variably/moderately/seriously

20. I have a tingling sensation in my legs (pins and needles) [5]	not at all/slightly/variably/moderately/seriously
21. I have chest pain[4]	not at all/slightly/variably/moderately/seriously

[1] In this study is grouped as “cardiovascular disorders”

[2] In this study is grouped as “gastrointestinal disordes”

[3] In this study is grouped as “urinary disorders”

[4] In this study is grouped as “musculoskeletal disorders”

[5] In this study is grouped as “skin and mucous membranes disorders”

[6] In this study is grouped as “ophtalmologic disorders’
